# WSES consensus guidelines on sigmoid volvulus management

**DOI:** 10.1186/s13017-023-00502-x

**Published:** 2023-05-15

**Authors:** Brian W. C. A. Tian, Gabriele Vigutto, Edward Tan, Harry van Goor, Cino Bendinelli, Fikri Abu-Zidan, Rao Ivatury, Boris Sakakushev, Isidoro Di Carlo, Gabriele Sganga, Ronald V. Maier, Raul Coimbra, Ari Leppäniemi, Andrey Litvin, Dimitrios Damaskos, Richard Ten Broek, Walter Biffl, Salomone Di Saverio, Belinda De Simone, Marco Ceresoli, Edoardo Picetti, Joseph Galante, Giovanni D. Tebala, Solomon Gurmu Beka, Luigi Bonavina, Yunfeng Cui, Jim Khan, Enrico Cicuttin, Francesco Amico, Inaba Kenji, Andreas Hecker, Luca Ansaloni, Massimo Sartelli, Ernest E. Moore, Yoram Kluger, Mario Testini, Dieter Weber, Vanni Agnoletti, Nicola De’ Angelis, Federico Coccolini, Ibrahima Sall, Fausto Catena

**Affiliations:** 1 Department of General Surgery, Singapore General Hospital, Singapore, Singapore; 2 Acute Care Surgery Unit, Department of Surgery and Trauma, Maurizio Bufalini Hospital, Cesena, Italy; 3 Department of Surgery, Radboud University Medical Center, Nijmegen, The Netherlands; 4 Department of Traumatology, John Hunter Hospital and University of Newcastle, Newcastle, NSW Australia; 5 The Research Office, College of Medicine and Health Sciences, United Arab Emirates University, Al Ain, UAE; 6 Professor Emeritus Virginia Commonwealth University, Richmond, VA USA; 7 Research Institute at Medical University Plovdiv, University Hospital St George, Plovdiv, Bulgaria; 8 Department of Surgical Sciences and Advanced Technologies “GF Ingrassia”, Cannizzaro Hospital, University of Catania, Catania, Italy; 9 Fondazione Policlinico Universitario A. Gemelli IRCCS, Catholic University, Rome, Italy; 10 Department of Surgery, Harborview Medical Center, University of Washington, Seattle, WA USA; 11 Division of Trauma, Surgical Critical Care, Burns, and Acute Care Surgery, Department of Surgery, UCSD Health System - Hillcrest Campus, San Diego, CA USA; 12 Department of Abdominal Surgery, Abdominal Center, University of Helsinki and Helsinki University Central Hospital, Helsinki, Finland; 13 Department of Surgery, Immanuel Kant Baltic Federal University, Kaliningrad, Russia; 14 Department of Upper GI Surgery, Royal Infirmary of Edinburgh, Edinburgh, Scotland, UK; 15 Queen’s Medical Center, University of Hawaii, Honolulu, HI USA; 16 Trauma and General Surgeon Royal Perth Hospital, The University of Western Australia, Perth, Australia; 17 Department of Minimally Invasive Surgery, Guastalla Hospital, AUSL-IRCCS Reggio, Emilia, Italy; 18 Emergency and General Surgery Department, University of Milan-Bicocca, Milan, Italy; 19 Department of Anesthesia and Intensive Care, Parma University Hospital, Parma, Italy; 20 Trauma Department, University of California, Davis, Sacramento, CA USA; 21 Department of Digestive and Emergency Surgery, S. Maria Hospital Trust, Terni, Italy; 22 School of Medicine and Health Science, University of Otago, Wellington Campus, Wellington, New Zealand; 23 Division of General Surgery, IRCCS Policlinico San Donato, University of Milan, Milan, Italy; 24 Department of Surgery, Nankai Clinical School of Medicine, Tianjin Nankai Hospital, Tianjin Medical University, Tianjin, China; 25 Department of Colorectal Surgery, Queen Alexandra Hospital, University of Portsmouth, Southwick Hill Road, Cosham, Portsmouth, UK; 26 General, Emergency and Trauma Surgery, Pisa University Hospital, Pisa, Italy; 27 Division of Trauma, Critical Care University of Southern California, Los Angeles, USA; 28 Department of General and Thoracic Surgery, University Hospital of Giessen, Giessen, Germany; 29 General Surgery Department, Papa Giovanni XXIII Hospital, Bergamo, Italy; 30 Department of Surgery, Macerata Hospital, Macerata, Italy; 31 Department of Surgery, Denver Health Medical Center, Denver, CO USA; 32 Division of General Surgery, Rambam Health Care Campus, Haifa, Israel; 33 Academic Unit of General Surgery “V. Bonomo”, Department of Biomedical Sciences and Human Oncology, University of Bari, Bari, Italy; 34 Department of General Surgery, Royal Perth Hospital, University of Western Australia, Perth, Australia; 35 Anesthesia and Intensive Care Unit, AUSL Romagna, M. Bufalini Hospital, Cesena, Italy; 36 Department of Digestive, Hepato-Pancreato-Biliary Surgery and Liver Transplantation, Henri Mondor University Hospital, Paris, France; 37 General Surgery Department, Military Teaching Hospital, Dakar, Senegal

## Abstract

Sigmoid volvulus is a common surgical emergency, especially in elderly patients. Patients can present with a wide range of clinical states: from asymptomatic, to frank peritonitis secondary to colonic perforation. These patients generally need urgent treatment, be it endoscopic decompression of the colon or an upfront colectomy. The World Society of Emergency Surgery united a worldwide group of international experts to review the current evidence and propose a consensus guidelines on the management of sigmoid volvulus.

## Background

The term “volvulus” comes from the Latin “volvere” meaning twist. It was first described by Rokitansky in 1836 [1]. Colonic volvulus is therefore the twisting of a segment of colon on its mesentery. Colonic volvulus is the third leading cause of colonic obstruction globally, following colorectal cancer and complicated sigmoid diverticulitis [2].

The incidence of colonic volvulus, however, does vary in different regions of the world. In the “volvulus belt,” an endemic area that includes Africa, South America, Russia, Eastern Europe, the Middle East, India and Brazil, colonic volvulus represents 13% to 42% of all intestinal obstructions [3–6]. Conversely, volvulus accounts for 10% to 15% of all large-bowel obstructions in the USA and Western Europe [7–10]. Halabi et al. [9] reported on 63,749 cases of colonic volvulus among 3,351,152 cases of intestinal obstruction over a 9-year period. During this period, the authors observed a stable incidence of sigmoid volvulus; however, the incidence of cecal volvulus increased by 5% per year.

Although any mobile segment of the colon can twist on itself; the sigmoid is involved in 60–75% of cases, cecum in 25–40% of cases, transverse colon in 1–4% of cases and splenic flexure in 1% of cases [11]. The clinical presentation of volvulus does appear to have some differences, depending on location. In countries in the “volvulus belt,” sigmoid volvulus usually occurs in young men (from the 4th decade onward with a male/female sex ratio of 4:1). In Western countries, sigmoid volvulus preferentially affects elderly males (age > 70) while cecal volvulus affects somewhat younger females (age ≤ 60), as highlighted in the study by Halabi et al. [9]. For this reason, some authors consider that endemic sigmoid volvulus is a different clinical entity than sporadic volvulus [12].

The etiology of colonic volvulus is probably multifactorial. Some factors are common to all locations, such as chronic constipation, high fiber diet, frequent use of laxatives and anatomic predisposition [11].

Dolicho-sigmoid, the presence of an elongated sigmoid colon on a narrow mesenteric base, is the most commonly cited predisposing factor for sigmoid volvulus. An anatomical study performed on 590 cadavers demonstrated ethnic anatomical differences [13]. The length and height of the sigmoid colon were significantly longer, and the root of the meso-sigmoid was much narrower in Africans, with no difference between men and women. In the case–control study of Akinkuotu et al. [10], there was a significant increase in the length of the meso-sigmoid, the maximum width of the meso-sigmoid and the luminal circumference of the colon in patients who underwent surgery for sigmoid volvulus. However, there was no significant difference in the width of the meso-sigmoid root. The authors concluded that the combination of a high and wide meso-sigmoid with a narrow root predisposed to sigmoid volvulus. While there were clear anatomical predispositions, it remains unclear whether they were congenital or acquired [14].

Other risk factors that may cause the development of sigmoid volvulus include diabetes, neuropsychiatric issues that potentially lead to reduced autonomy, institutional placement and prolonged bed rest. Finally, in younger patients, some cases of sigmoid volvulus may be associated with megacolon, which in turn are due to causes such as Hirschsprung’s or Chagas disease [3].

In sigmoid volvulus, meso-sigmoid twisting of up to 180° is considered physiological. In approximately 2% of cases, the volvulus reduces spontaneously [15]. Torsion beyond 180° can lead to complications such as colonic obstruction, ischemia or necrosis with perforation. For unknown reasons, the twist preferentially occurs in the counterclockwise direction in 70% of cases [16].

Fibrosis of the meso-sigmoid, seen in 86% of operated patients, is more a result than a cause of the torsion. This cicatrization most likely occurs as a result of reversible ischemia, which can occur in the relapsing forms of volvulus [17]. The mechanics of this ischemia is thought to occur as follows. When sigmoid volvulus occurs, the subsequent colonic distension causes an increase in intraluminal pressure, which results in decreased capillary perfusion. This mural ischemia is further aggravated by meso-colic vessel occlusion, which is caused by the mechanical compression and axial rotation of the volvulus [18].

Early mucosal ischemia promotes bacterial translocation and bacterial gas production, further increasing colonic distension and toxic phenomena. If colonic torsion is not promptly reversed, this creates a vicious circle leading to colonic necrosis and ischemia–reperfusion injury. The two main mechanisms of torsion in sigmoid volvulus are believed to be either axial meso-colic volvulus (75% of the time) or organo-axial volvulus (25% of the time) [19].

Sigmoid volvulus typically presents in patients > 60 years old and typically has recurrent presentations, with each episode potentially bearing significant morbidity and mortality [3, 5, 20]. The management includes relief of the volvulus either by endoscopic or operative means, assessment of the viability of the volved colonic segment and preventing recurrence of the problem. Without definitive operative treatment, colonic volvulus tends to recur, with each episode presenting a risk of ischemia and perforation [2, 21–24].

Therefore, the aim of this paper was to perform a review of the existing literature and to provide recommendations on the management of sigmoid volvulus. These guidelines were reviewed by an international expert panel composed of 34 experts who were asked to critically revise the manuscript and recommendations. These guidelines were produced according to the World Society of Emergency Surgery (WSES) methodology. We shall present the derived statements upon which a consensus was reached, specifying the quality of the supporting evidence and suggesting future research directions.

## Purpose and use of these guidelines

These guidelines are evidence-based, with the grades of recommendation based on the evidence. They do not represent the standard of practice, but are suggested plans of care, based on best available evidence and a consensus of experts. They do not exclude other approaches as being within a standard of practice. The treating clinician should determine the most appropriate action, after taking into account conditions at the relevant medical institution (staff levels, experience, equipment, etc.) and the characteristics of the individual patient. The responsibility for the management and outcome rests with the engaging practitioners, and not the consensus group.

## Methods

An organized search of relevant literature was performed using the following databases: PubMed, Ovid MEDLINE, Embase, the Cochrane Database of Systematic Reviews, the Cochrane Central Register of Controlled Trials and the National Guidelines Clearinghouse (www.guideline.gov). Retrieved literature was not limited to the English language.

The terms sigmoid volvulus, volvulus, malrotation, intestine torsion, intestinal volvulus, decompression, colectomy, resection, imaging, Hartmann’s, megacolon, pseudo-obstruction, Ogilvie’s and follow-up in various combinations with the use of the Boolean operators “AND” and “OR.” No search restrictions were imposed. Clinical trials, consensus conferences, comparative studies, congresses, guidelines, government publications, multicenter studies, systematic reviews, meta-analyses, large case series, original articles, randomized controlled trials, case reports and small case series were included. We also analyzed the reference lists of relevant narrative review articles identified during the search to identify any studies that may have been missed.

Prospective, randomized controlled trials and meta-analyses were given preference in developing these guidelines. The final grade of recommendation was performed using the Grades of Recommendation, Assessment, Development, and Evaluation system.

The literature search and selection were performed by two reviewers (BT and GV). First, all records from merged searches were reviewed for relevance concerning the title and abstract. Records were removed when both reviewers excluded them. Both reviewers then performed an independent full-text analysis, which allowed to finally include or exclude the preselected article.

## Recommendations

**Recommendation 1: Initial evaluation should include a focused history and physical examination. A full panel of blood tests including blood gas and lactate levels are also important to look for suggestions of bowel ischemia. Grade of recommendation: Strong recommendation, based on low- or very-low-quality evidence, 1C.**

Symptoms of sigmoid volvulus include abdominal pain, constipation and vomiting (a late sign) [4–6, 9, 19, 27, 28]. In 30–41% of cases, patients report previous episodes of abdominal distention [3]. This triad is much more common in endemic volvulus, rather than the sporadic kind of volvulus (88% versus 33%) [28].

In the “volvulus belt” countries, the clinical presentation may be acute with peritonitis and shock. In this fulminant clinical presentation, the prognosis is poor because colonic necrosis and perforation would possibly have already occurred, by the time the patient first presents for care [25]. Conversely, in Western countries, the patient usually presents 3 to 4 days after the onset of symptoms [26]. The classic patient is elderly, institutionalized and under psychotropic medications that causes chronic constipation. The history should elicit the above-mentioned risk factors, including a personal history of previous sigmoid volvulus, which is present in 30–40% of cases.

Classically, the clinical examination will identify abdominal distension, diminished bowel sounds and often an empty rectum on digital examination [2, 20, 27–29]. However, the examination is often difficult due to the presence of abdominal distension, which is the result of colonic obstruction of several days duration; if there are no signs of peritoneal irritation, as is often the case, this may result in a delay in diagnosis. Nonetheless, the absence of peritonitis does not indicate the absence of bowel ischemia. Asymmetric gaseous abdominal distention associated with emptiness of the left iliac fossa is pathognomonic for sigmoid volvulus, though this can be very challenging to detect [5].

The duration of symptoms lasts from a few hours to several days [5, 20, 21, 26–28, 30–32]. As these patients are typically old with comorbidities, any vomiting and dehydration can tip them over into renal insufficiency. Thus, blood testing of electrolytes and renal function is necessary.

Bear in mind that as these patients may have neuropsychiatric issues, history may not be forthcoming or accurate. The physical examination and testing of blood gas and lactate levels are crucial, although bowel ischemia may be present in the absence of hyperlactatemia.

**Recommendation 2: Diagnostic imaging for sigmoid volvulus is initially based on plain abdominal radiographs, showing a classic coffee bean sign. Grade of recommendation: Strong recommendation, based on low- or very-low-quality evidence, 1C.**

Plain abdominal radiographs are often diagnostic of sigmoid volvulus. Chest radiographs are also sufficient to detect the presence of free air, in cases of perforation. Imaging should be done expediently. The classic finding is that of a coffee bean, projecting toward the upper abdomen, sometimes above the transverse colon, which has been described as the “northern exposure sign” [5, 29, 33–37].

**Recommendation 3: CT imaging can be used in cases where the diagnosis is in doubt, or if ischemia or perforation is suspected. Grade of recommendation: Strong recommendation, based on low- or very-low-quality evidence, 1C.**

In cases in which clinical assessment and plain abdominal radiographs are insufficient to confirm the diagnosis of sigmoid volvulus, or if a complication is suspected (e.g., impending ischemia), urgent CT imaging is indicated. When performing CT imaging, the use of intravenous contrast can facilitate the diagnosis of colonic ischemia [35, 36, 39–41]. In the study by Swenson et al. [21], the positive diagnostic yield of CT for sigmoid volvulus was 89%. Other diagnoses that can mimic the presentation of colonic volvulus, such as obstruction due to a neoplasm or pseudo-obstruction, can also be evaluated with the above modalities.

The addition of a contrast enema may help confirm the diagnosis of sigmoid volvulus by demonstrating a “bird’s beak” sign, at the point of colonic torsion [5, 24, 28, 33, 37, 38]. However, an enema is strictly contraindicated when perforation is suspected. When using a contrast enema, a water-soluble contrast is much preferred over barium contrast, because the latter could cause a chemical peritonitis in the setting of a perforated colon.

**Recommendation 4: In patients in whom ischemia or perforation is not suspected clinically and/or radiologically, flexible endoscopy should be performed as a first line to decompress the sigmoid colon. Grade of Recommendation: Strong recommendation, based on low- or very-low-quality evidence, 1C.**

In the absence of colonic ischemia or perforation, the initial treatment of sigmoid volvulus is urgent endoscopic detorsion, which is effective in 60–95% of patients [3, 21, 22, 27, 42–44]. Endoscopic detorsion carries a 4% morbidity, and some series show a 3% mortality rate [22, 27, 45].

Successful detorsion implies that the endoscopist must visualize and go past the transition points (typically 2 points are found) [2, 20, 22, 45, 46]. At the end of detorsion, endoscopic view of the mucosa to assess sigmoid colon viability is mandatory**.** After successful detorsion of the sigmoid colon, a decompression flatus tube should be left in place to maintain the reduction, allow for continued colonic decompression, and facilitate mechanical bowel preparation as needed [2, 20–22, 43, 44, 47–52].

After successful endoscopic detorsion, long-term recurrence has been observed in 43% -75% of patients [20–22, 26, 47, 52, 53]. As each future episode of volvulus carries its attendant risks of ischemia or perforation, operative intervention should be strongly considered during the index admission or soon thereafter [20–22, 26, 52, 54].

The literature favors flexible endoscopy over rigid endoscopy because of its superior diagnostic performance, particularly in assessing ischemia and because of its lower perforation rate [36]. Rigid sigmoidoscopy can fail to diagnose sigmoid volvulus and miss ischemia in up to 24% of cases.

The favorable impact of colonoscopy is perfectly illustrated in Turkey’s very large retrospective series that compiled 952 patients, over a period of 46.5 years [22]. Colonic decompression had evolved from the initial use of barium enema (1966–1968), to rigid sigmoidoscopy (1968–1988), to the introduction of the flexible endoscope in 1988, and exclusive use of flexible endoscopic decompression from 2003 onwards. In the Turkish experience, barium enema resulted in successful decompression in 69% of cases but was burdened with a morbidity of 23%, a mortality of 8% and early recurrence in 11% of cases. With rigid sigmoidoscopy, the authors observed successful decompression, morbidity, mortality and early recurrence rates of 78%, 3%, 1% and 3%, respectively. With the advent of flexible endoscopy, rates of successful decompression, morbidity, mortality and early recurrence were 76%, 2%, 0.3% and 6%, respectively.

Yassaie et al. [47] described 31 patients with sigmoid volvulus who underwent successful endoscopic detorsion and no further interventions. Recurrent volvulus was diagnosed in 19 (61%) of these patients at a median of 31 days. Of these 19 patients, 7 underwent colectomy and 12 had repeat endoscopic detorsion alone, of whom 5 (48%) were diagnosed with a third episode of volvulus at a median interval of 5 months and 3 (25%) required emergent sigmoid colectomy [47].

Nonetheless, in cases in which advanced mucosal ischemia, perforation or impending perforation of the colon are discovered during endoscopy, the procedure should be aborted. Emergency colectomy is warranted in these cases.

There seems to be little role for a completion screening colonoscopy before surgery, mainly because of its technical difficulty. The colon is often extremely long and redundant, with angulations that are difficult to traverse. Preoperative total colonoscopy should be offered only if there is clinical or radiological suspicion of underlying neoplasia [21, 55, 56].

Endoscopy is therefore limited in most cases to short flexible colonoscopy performed during endoscopic detorsion, which also rules out neoplastic obstructions at the rectosigmoid junction, the other principal entity in the differential diagnosis. In case of diagnostic uncertainty, a virtual colonography can be performed instead of total colonoscopy.

**Recommendation 5: Urgent sigmoid resection is indicated when endoscopic detorsion of the sigmoid colon is not successful and in cases of non-viable or perforated colon. Strong recommendation, based on low- or very-low-quality evidence, 1C.**

In 5–25% of patients with sigmoid volvulus, they will present with colonic ischemia, perforation, peritonitis or septic shock on admission. These patients require upfront urgent colectomy [2, 4, 20–22, 26, 27, 42, 49, 57–61]. Intraoperatively, resection of infarcted bowel should be performed without detorsion and with minimal manipulation to prevent release of endotoxin, potassium and bacteria into the general circulation and to avoid perforation of the colon [24, 51, 62–64].

The decision to perform an isolated sigmoid colectomy versus a high anterior resection should be individualized. However, since this is a benign pathology, a full oncological anterior resection is not typically needed. The main consideration would be the vascular supply of the remnant colon. The decision to perform primary colorectal anastomosis, defunctioned colorectal anastomosis or end colostomy should be individualized, with consideration of both the overall condition of the patient and the colon.

Kuzu et al. [60] reported on 106 sigmoid volvulus cases accumulated over 8 years. They performed sigmoid resection with end colostomy (Hartmann procedure, *n* = 49) or sigmoid resection with colorectal anastomosis without diverting ostomy (*n* = 57). A Hartmann procedure was used more often in patients with a non-viable colon or peritonitis and resulted in increased postoperative complications and mortality (8% vs 5%), whereas anastomotic leak occurred in 7% of patients in the anastomosis group [60].

Atamanalp et al. [20] reported on 952 patients with sigmoid volvulus. In this series, a Hartmann procedure was the most commonly performed emergency operation, with overall morbidity of 42% and mortality of 20%. Coban et al. [60] reported on sigmoid resection with non-diverted or diverted colorectal anastomosis and found 12% and 0% anastomotic leaks and a mortality rate of 8% and 10%, respectively.

Overall, there are insufficient data to support one technique over another in emergent cases for sigmoid volvulus, as most show no difference in mortality or overall surgical postoperative complications among the various approaches [57–59, 65, 66]. Despite the evidence, end colostomy creation is often the most appropriate choice for hemodynamically unstable patients or when there are significant concomitant factors, such as increased ASA or Acute Physiology and Chronic Health Evaluation II score, coagulopathy, acidosis or hypothermia, all of which add prohibitive risk to the integrity of a colorectal anastomosis [22, 58, 60, 67–69].

The role of laparoscopic surgery for emergency colorectal operations is still unclear. One recent comparison of open and laparoscopic cases demonstrated a twofold increase in anastomotic leaks in the latter group but similar overall postoperative morbidity [57]. Additional published results indicate that the laparoscopic approach is a suitable alternative to laparotomy in select cases by surgeons who are competent with this technique [34, 70–72].

**Recommendation 6: For patients with successful endoscopic decompression, sigmoid colectomy should be offered to prevent recurrent volvulus. The colectomy should be performed as early as possible, even during the index admission. Grade of Recommendation: Strong recommendation based on low-quality evidence, 1C.**

After colonoscopic detorsion followed by conservative management, the recurrence rate of sigmoid volvulus varies from 45 to 71% [21, 27, 49, 55, 56]. This tendency persists in recently published studies both in France (67% in the experience of the Saint-Antoine hospital [26]), Turkey (nearly 2 out of 3 patients with follow-up exceeding 40 years), New Zealand (61% at 3 months [47]) or in the Danish registry with recurrence probability of 63%, 47%, 41% and 24%, respectively, at 3, 6, 12 and 24 months [73]. In addition, the mortality after conservative treatment in the literature varies between 9% and 36% [21, 27, 49, 55, 56]. In the Danish registry, survival was significantly lower after conservative treatment [53]. However, these results must be qualified since patients considered non-surgical from the start were significantly older and had a significantly higher ASA score (82 vs. 71, *P* = 0.004; ASA 3 vs. ASA 2, *P* = 0.012).

In the absence of a randomized study, the current consensus is to perform colonic resection within the index admission of the first episode of sigmoid volvulus, because of the high risk of recurrence [54].

Sigmoid colectomy is the intervention that is most effective at preventing recurrent volvulus [2, 20, 22, 26, 30, 47, 51, 70, 74]. The entire length of the redundant colon should be removed. The non-urgent sigmoid resection results in low morbidity and mortality in the range of 0–12% [2, 20, 26, 27, 47, 52]. The decision for laparotomy versus laparoscopy should be left to the comfort of the surgeon [20, 70, 71]. Typically, stoma creation in the non-emergency setting is not usually required.

**Recommendation 7: Non-resectional operative procedures (detorsion, sigmoidoplasty and mesosigmoidoplasty) are inferior to sigmoid colectomy for the prevention of recurrent volvulus and should be avoided. Strong recommendation based on low-quality evidence, 1C.**

Operative detorsion alone, detorsion with intraperitoneal or extraperitoneal fixation (sigmoidopexy) and tailoring of the sigmoid mesentery to broaden its base and prevent torsion (mesosigmoidopexy) are non-resectional techniques that have been described for the definitive treatment of sigmoid volvulus in patients with a viable colon. Recurrence after the non-resectional techniques is variable, but expectedly higher than a sigmoid resection [2, 5, 20, 22].

Bhatnagar and Sharma [75] performed detorsion and extraperitoneal sigmoid colon fixation in 84 patients in whom no recurrences were observed. However, other series have described a 29–36% recurrence rate after sigmoidopexy [4, 26, 50].

Subrahmanyam [76] achieved excellent results with mesosigmoidoplasty. They had recurrence in only 2 out of 126 patients. Akgun [77] reported no recurrence in 15 patients after mesosigmoidoplasty. However, Oren et al. [22] and Atamanalp [20] reported a 16–21% recurrence rate after mesosigmoidoplasty.

Studies have shown that detorsion only results in 30–35% morbidity and 11–15% mortality. It also has a recurrent volvulus rate of 18–48%. This method of intervention is now discouraged [20, 22, 25, 51, 52].

**Recommendation 8: Endoscopic fixation of the sigmoid colon may be considered in select patients in whom operative interventions present a prohibitive risk. Grade of Recommendation: Weak recommendation based on low-quality evidence, 2C**.

Sigmoid volvulus is often encountered in older patients, some of whom may be unfit for abdominal operations. For this subset of patients, small case series have reported advanced endoscopic techniques as a less invasive means to prevent recurrent sigmoid volvulus.

The percutaneous endoscopic colostomy (PEC) technique is performed to fix the sigmoid colon to the anterior abdominal wall, restricting its mobility, with the aim of preventing recurrent volvulus. Fixation of the colon has been performed using T fasteners or by percutaneous tube colostomy placement with or without laparoscopic assistance [26, 78–83].

Baraza et al. [78] performed PEC on 19 elderly patients with recurrent sigmoid volvulus. Baraza found major complications (including peritonitis, tube migration and death) occurred in 2 patients (10%) and minor complications (e.g., abdominal wall bleeding or infection) occurred in 7 patients (37%). There were 8 deaths from unrelated causes. Of the 6 patients who underwent removal of the PEC tube(s), after 6 to 26 months of fixation, none experienced recurrent volvulus at a median follow-up of 35 months.

Daniels et al. [79] reported on 14 patients with PEC. The PEC maintained reduction of the volvulus in each of the 5 patients in whom it was left in place but in only 3 of 6 in whom the PEC was subsequently removed. At present, PEC should generally be reserved for patients in whom established operative interventions are deemed too high risk.

**Recommendation 9: Patients who have concomitant megacolon and sigmoid volvulus, should undergo subtotal colectomy. Sigmoid colectomy alone is insufficient as the volvulus tends to recur in the remnant segments of colon. Grade of Recommendation: Strong recommendation based on low-quality evidence, 1C**.

Sigmoid volvulus in association with megacolon is not a well-published area of research. Clinically, this condition is suspected when a digital rectal examination reveals a capacious rectum and the colon proximal to the volvulus is dilated significantly throughout [84]. These patients suffer from chronic colonic constipation and dysfunction [85–88]. Limited resection of the sigmoid will not be adequate. Intraoperative findings of concomitant megacolon and/or megarectum will predict for increased recurrence [89].

Morrissey et al. [87] reviewed the long-term postoperative course of 29 patients who underwent surgery for sigmoid volvulus. The overall recurrence rate was 36%. The major variable was the degree of colonic involvement, since patients whose disease was limited to the sigmoid colon had a 6% recurrence rate compared to 82% for those with associated megacolon (*p* = .005). In patients with megacolon treated by subtotal colectomy, no recurrences were documented.

Strom et al. [88] reviewed a 30-year experience in management of 129 patients with 163 acute obstructions due to sigmoid volvulus. Recurrent obstruction of the colon was observed in 47 (or 45%) of 104 patients who survived their initial obstructive episode. Sigmoid volvulus was identified to be the cause of recurrent obstruction in 36 of 47 patients, while atonic bowel, involving the sigmoid alone or more proximal colon as well, was responsible for the other 11 recurrent obstructions. Strom concluded that sigmoid excision was corrective only if bowel atony was limited to that portion of the colon. Only more extensive colectomy, so as to include all flaccid colon, consistently obviated recurrence.

**Recommendation 10: Colonic volvulus in pregnancy is rare. Treatment will require a multidisciplinary approach, taking into account the stage of pregnancy. Grade of Recommendation: Strong recommendation based on low-quality evidence, 1C.**

Colonic volvulus is the first or second leading cause of organic bowel obstruction in pregnant women, although very few cases have been reported in the literature (about a hundred). Both diagnosis and treatment pose problems that may threaten both the maternal and especially, the fetal prognosis. It typically occurs in a multiparous woman (in 75% of cases), and in the 3rd trimester in two-thirds of cases [90].

The clinical and laboratory abnormalities are non-specific. Maternal and fetal prognosis is both worsened by delay in diagnosis that can lead to colonic necrosis in 23% of cases [91]. Choice of imaging modalities depends on the term of the pregnancy but magnetic resonance imaging may be an attractive option [92]. For uncomplicated sigmoid volvulus, endoscopic detorsion is recommended but may be ineffective especially in the third trimester because of the volume of the uterus.

The multidisciplinary strategy will therefore depend on the term of pregnancy and the fetal prognosis. In ideal circumstances, definitive surgery is recommended after childbirth, but can be performed without significant impact on the fetus, from the second trimester onward.

The reported rates of maternal and fetal mortality are 6–12% and 20–26%, respectively [92].

**Recommendation 11: Ileosigmoid volvulus is rare and most require surgical decompression. Grade of Recommendation: Strong recommendation based on low-quality evidence, 1C**.

Ileosigmoid volvulus is exceptional, although near endemic in the “volvulus belt” of Africa, Asia and the Middle East. Affected patients are usually young men (4th to 5th decade) [93].

Three types of ileosigmoid volvulus have been described:

- Type I: the ileum wraps around the sigmoid clockwise or anticlockwise (about 55% of cases);
- Type II: sigmoid wraps around the ileum clockwise or counterclockwise (about 5% of cases);
- Type III: the ileocecal region wraps around the sigmoid (less than 5% of cases).

There are some unclassifiable variants; the rotation is clockwise in about 2/3 of cases [93].

The clinical picture is that of an acute onset of bowel obstruction, often with systemic toxicity. Unfortunately, there is often treatment delay. Indeed, the diagnosis is made in only 20% of cases and intestinal necrosis of the ileum and/or sigmoid colon is observed in 70% of cases. Diagnosis currently relies on abdominopelvic CT. The therapeutic management requires fluid and electrolyte resuscitation followed by surgery: double resection with or without restoration of continuity depending on the operating findings. Mortality is high, reaching 73% in some series [94].

## Discussion

Sigmoid volvulus is the third most common cause of large-bowel obstruction [27]. It has a wide geographic variation, and it differs significantly between high-incidence countries and low-incidence countries [15]. This variation may be associated with differences in anatomy [10]. Acute sigmoid volvulus usually occurs in adult men. The mean age was found to be between 56 and 77 years, and nearly one-third of all colonic emergencies in elderly patients are due to sigmoid volvulus [95].

Sigmoid volvulus can cause a wide range of symptoms from non-specific abdominal pain to acute abdomen. A proper patient assessment has to focus on clinical history, physical examination and blood tests to discern between critical patients and non-critical ones [2, 3, 5, 20, 21, 27, 28, 30–32].

Urgent radiology is critical in achieving a diagnosis. Plain abdominal radiographs are the first line tests. The classical sign of sigmoid volvulus is the coffee bean sign. Abdominal CT remains the gold standard and usually reveals a dilated colon with an air/fluid level and the “whirl sign,” which represents twisted colon and mesentery [5, 21, 24, 28, 33–41, 95].

Raveenthiran et al. [5] recently provided more insight into the pathophysiology of acute sigmoid volvulus. Increasing intraluminal pressure impairs capillary perfusion following the occurrence of acute sigmoid volvulus. Mechanical obstruction due to twisting of mesenteric vessels and thrombosis of meso-sigmoid veins contribute to ischemia. Ischemic injury in the mucosa occurs earlier than in other colonic layers and facilitates bacterial translocation and toxemia. A competent ileocecal valve converts the proximal colon into a second “closed loop.” Prompt and optimal correction of these pathophysiological features is vital to improve the prognosis of sigmoid volvulus.

The optimal treatment of sigmoid volvulus depends on the patient’s initial presentation. If the patient presents with septic shock or bowel ischemia or perforation, an urgent upfront surgery is warranted. Performing a single-step resection and anastomosis or a Hartmann’s procedure should be based on the patient’s overall clinical condition and intraoperative findings, e.g., presence of abdominal fecal contamination. The data on the benefits of a laparoscopic approach in the emergency setting as compared to an open approach still remain unclear [2, 4, 20–22, 26, 27, 42, 49, 57–61, 65–72].

Emergency surgery is associated with significant mortality and morbidity. Kassi et al. [96] reported that the mortality rate was 12% (*n* = 3) for Hartmann’s procedure. Surgical site infections (42.86%) were the most common complications. 11 of 22 (50%) patients had intestinal continuity restored. Bhatnagar et al. [58] reported that the risk factors for mortality were: (1) age over 60 years; (2) presence of shock on admission; and (3) positive history of a previous episode of volvulus. With regard to the former two risk factors, special efforts are necessary by intensive care staff to monitor homeostatic disturbance and reduce mortality in older patients (> 60 years) and those presenting with shock at the time of admission.

Conversely, if the patient is not in extremis, and the volvulus is uncomplicated, then the first line of treatment is endoscopic decompression [2, 3, 20–22, 26, 27, 47, 52–54]. We strongly recommend that after resolution of the volvulus, sigmoid resection should be offered and preferably performed during the index admission. Without resection, the change of a recurrence remains high [2, 4, 5, 20, 22, 26, 50, 76, 77] and quality of life may be impaired. In high-risk patients, endoscopic fixation of the colon (percutaneous endoscopic colostomy) can be considered [26, 78–83].

Non-operative treatment is successful in 70–91% of cases, with reported complication rates of 2–4.7% in geriatric patients [96, 97]. Colonoscopic derotation simply converts an emergency into an elective procedure, which facilitates treatment of comorbidity and allows preparation of the bowel prior to definitive surgery.

Following derotation, ischemia–reperfusion injury aggravates intestinal dysfunction, and even intestinal ulcer and perforation. Peritoneal exudate, high intestinal fluid accumulation, electrolyte disturbances and hypoproteinemia lead to serious adverse consequences. Consequently, effective treatment following colonoscopic derotation is very important. Fluid resuscitation should be performed immediately. Broad-spectrum antibiotics are indicated to control bacterial translocation across the ischemic intestinal wall [23].

## Conclusions

Sigmoid volvulus is a common emergency, especially in elderly patients. Urgent endoscopic decompression is warranted, except in cases where ischemia or colonic perforation is suspected, in which case upfront sigmoid colectomy is recommended. For patients who have had successful endoscopic decompression of the colon, an early elective resection with or without anastomosis should be considered to prevent future recurrence.

## Data Availability

Not applicable.
